# Cultural mode and organo-mineral amendment effect on growth and yield of rice (*Oryza sativa* L.) and soil chemical properties in sulfated acid soils of Basse-Casamance

**DOI:** 10.1016/j.heliyon.2023.e18830

**Published:** 2023-08-04

**Authors:** Abdoulaye Badiane, Barthélémy Arnold Faye, Antoine Sambou, Ismatou Ba, Kollé Diop, Mariama Diallo, Siny Gueye, Baboucar Bamba, Saliou Fall

**Affiliations:** a Institut Sénégalais de Recherches Agricoles (ISRA)/Centre de Recherches Agricoles (CRA) de Djibélor, Senegal; b Department of Agroforestry Assane Seck University of Ziguinchor, Senegal; c Institut Sénégalais de Recherches Agricoles (ISRA)/Laboratoire National de Recherches sur les productions Végétales, Senegal

**Keywords:** Rice, Performance, Yield, Cultural mode, Fertilizer, Salinity, Acidity

## Abstract

Climatic variability and the scarcity of rainfall have intensified the process of soil salinization, leading to land degradation and loss of rice yield. A field experiment was conducted to study the effect of cultural mode and organo-mineral fertilizers on rice performance and soil chemical properties. A split plot design with four replications and two factors that were cultural mode (flat and ridge) and fertilizers (mineral, organic, organo-mineral, and control) was carried out. Observations on growth, yield parameters, and yield of rice and soil chemical properties (pH and EC) were recorded. The cultural mode influenced significantly rice performance. Height (76.26 cm), tillers (89.93 m^-2^), panicles (71.66 m^-2^), biomass (3252.25 kg ha^−1^), 1000 kernel weight (12.85 g) and yield (1123.14 kg ha^−1^) were significantly higher in ridge than flat. However, infertility (44.74%), sterility (58.04%), and survival (91.86%) were higher in flat than ridge mode. However, sowing of rice on ridges with mineral and organo-mineral amendments improved yield parameters increasing the yield of rice more than in flat mode. Soil chemical properties were significantly influenced by cultural modes and fertilizers. Ridge mode increased the soil pH and reduced the salinity more than in flat. Organic and organo-mineral fertilizers affected significantly the soil's chemical parameters by improving the pH and reducing the salinity. Ridge mode combined with organo-mineral amendment improved rice performance and soil chemical properties. Cultural modes and fertilizer types were critical elements to improve soil pH, salinity, and yield.

## Highlights of the study

• Ridge cultivation favored rice performance while improving soil chemical properties (increasing soil pH and decreasing soil salinity).
• The various mineral, organic and organo-mineral fertilizers substantially improved rice yield and soil chemistry.
• Both organo-mineral and organic fertilizers significantly increased rice yield.
• Ridge mode combined with organo-mineral amendment improved rice performance and soil chemical properties.

## Graphical abstract

**Figure:**
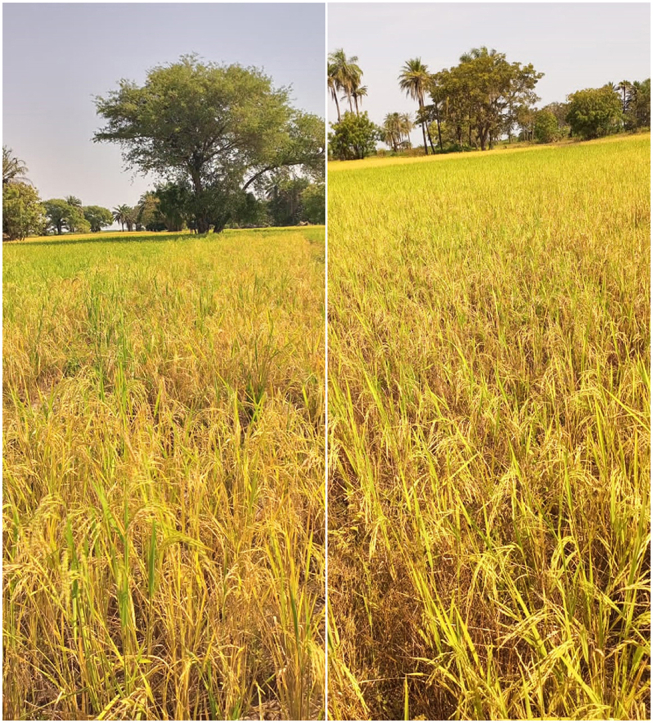

## 1 Introduction

Rice is eaten by about 3 billion people and is the most common staple food of the largest number of people on earth [1]. Rice (*Oryza sativa* L.) is a popular cereal in the world, particularly in Africa. Its consumption on the continent increased from 16 to 29 million tons between 2000 and 2012 and 12 to 24 million tons in Sub-Saharan Africa [2]. In Senegal, rice is among the most consumed cereals, and per capita consumption is estimated at 87.6 kg/year [3].

Despite its importance in the life of the populations, rice production is confronted with several anthropic and edaphic constraints (salinization and acidification). Soil acidity and salinity are critical constraints to crop production [4]. Soil salinity is one of the abiotic stresses that constrain rice crop growth. It affects at least 400 million hectares of land and seriously threatens an equivalent area [5]. Saline soils are mainly located in the arid climate zones of Africa [6], where about 38 million hectares of land, or 2% of the continent's surface area, are affected by salt. The problem first became apparent in Senegal in the 1920s as a result of worsening climatic conditions [7]. The phenomenon of salinization associated with acidification leads to a decrease in the productivity of cultivable land, up to the abandonment of fields, and threatens more than 50% of cultivable land in Senegal [8]. Acidification caused problems associated with decreased yields of rice grown on sulfated soils acid by adverse effects of H^+^, toxicity, electrolyte stress, CO_2_ and organic and inorganic acids, and impaired microbial activities [9]. Acid sulphate soils are a group of these soils having high soil acidity and other soil function limitations. They are characterized by a low pH and the presence of sulphidic materials and/or a sulphuric horizon [10].

In response to this situation, management strategies for these soils involving green manuring, rice cultivation, and application of fertilizers before sowing were needed. Control strategies based on the construction of hydro-agricultural structures (anti-salt dams), chemical (phosphogypsum) and organic amendments, and the use of rice varieties (Warr 77, Warr 1, Rock 5, and ISRIZ 10) have been developed by researchers and adopted by farmers. It would be important to combine compost with mineral additives under a suitable cropping pattern. Some authors believe that the addition of mineral additives acts on the properties of compost to release the necessary nutrients to the plant [[11], [12], [13]]. This study aimed to evaluate the effects of cultural modes and organo-mineral fertilization on rice performance and soil chemical properties.

## 2 Material and methods

### 2.1 Study area

**Fig. 1:**
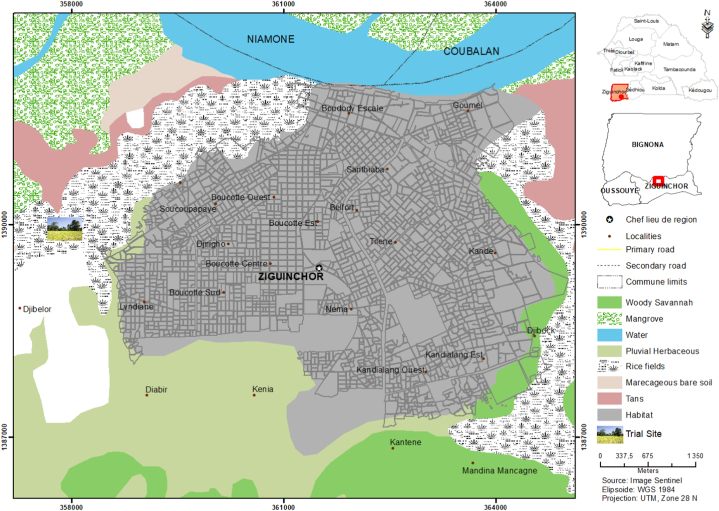
Study area location.

The trial was conducted at the new rice station of ISRA/CRA of Djibélor (12°56′16″ North and 16°30′60″ West) located in the commune of Niaguis southwest of Ziguinchor, Senegal (Fig. 1). The climate of the region is of the coastal South Sudanian type [14]. It was characterized by the existence of two seasons: a dry season from November to May and a rainy season from June to October [15]. The average temperature for the year 2021 was approximately 35 °C. The annual cumulative rainfall was estimated at 990 mm.

### 2.2 Experimental design and treatments

**Fig. 2:**
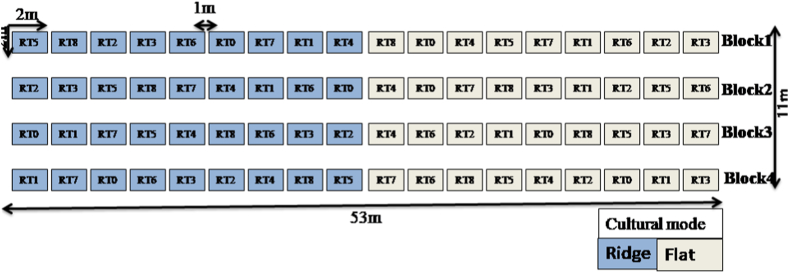
Experimental design. R:Ridge; F:Flat, T0: control, T1: 100 kg ha^−1^ NPK +75 kg ha^−1^ N, T2: 200 kg ha^−1^ NPK +150 kg ha^−1^ N, T3: 5 t ha^−1^ compost, T4: 5 t ha^−1^ compost +100 kg ha^−1^ NPK +75 kg ha^−1^ N, T5: 5 t ha^−1^ compost +200 kg ha^−1^ NPK+150 kg ha^−1^ N, T6: 7.5 t ha^−1^ compost, T7: 7.5 t ha^−1^ compost +100 kg ha^−1^ NPK +75 kg ha^−1^ N, T8: 7.5 t ha^−1^ compost +200 kg ha^−1^ NPK+ 150 kg ha^−1^N.

A split plot design with four replications or blocks and two factors that were cultural mode (flat and ridge) and fertilizers (mineral, organic, organo-mineral, and control) was carried out (Fig. 2). Each block is 53 m long and 2 m wide with a 1 m separation between them. Each block is subdivided into 18 elementary plots (2 m *2 m) corresponding to each other. The fertilizers are organic (compost), mineral (NPK and N), and organo-mineral (compost + NPK + N) with different doses and a control (no input) with nine levels (Table 1). The compost used was composed of ash, rice straw, mango leaves, potting soil, male *Elaeis guineensis* flowers and cow dung. The combination of the two factors (cultural mode and fertilization) allowed us to obtain 18 treatments (Fig. 2). Compost and triple (NPK 15-15-15) were applied and run off three days after the installation of the system, i.e. 10 days before transplanting. Urea (46% N) was applied twice at 15 and 60 days after sowing. The rice variety “ISRIZ 10” was used for transplanting adopted in irrigated and lowland systems for cultivation duration of 122 to 130 days [16]. Transplanting of seedlings was carried out at a rate of 100 plants per elementary plot with a spacing of 0.2 m * 0.2 m. Yield squares of five rows were established in each elementary plot to collect agro-morphological data. The field experiment was conducted during the pluvial season 2021.

**Table 1:** Different fertilizers and their composition.

Fertilizers	Composition
T0	Control (without amendment)
T1	100 kg ha^−1^NPK (15-15-15) + 75 kg ha^−1^ N (46-00-00)
T2	200 kg ha^−1^ NPK (15-15-15) + 150 kg ha^−1^N (46-00-00)
T3	5 t ha^−1^ compost
T4	5 t ha^−1^ compost + 100 kg ha^−1^ NPK (15-15-15) + 75 kg ha^−1^ N (46-00-00)
T5	5 t ha^−1^ compost + 200 kg ha^−1^ NPK (15-15-15) +150 kg ha^−1^ N (46-00-00)
T6	7.5 t ha^−1^ compost
T7	7.5 t ha^−1^ compost + 100 kg ha^−1^ NPK (15-15-15) + 75 kg ha^−1^ N (46-00-00)
T8	7.5 t ha^−1^ compost + 200 kg ha^−1^ NPK (15-15-15) + 150 kg ha^−1^N (46-00-00)

### 2.3 Data collection

Observations and measurements were made to determine the agro-morphological parameters and soil chemical properties.

#### 2.3.1 Growth and yield parameters and yield

Growth (height and recovery) and yield (sterility, infertility, tillers, and panicles) parameters and yield (1000 grains, biomass, and yield) were measured or determined. Survival consisted of counting plants that survived 15 days after transplanting in each elementary plot to determine the survival rate. Plant height was measured on eight randomly selected plants in the yield square on 60 days after transplanting. The total number of tillers and panicles per square meter (m^2^) before the harvest was counted on four plants taken at random from the yield square. The number of infertile tillers was counted to determine the infertility rate. After harvest, infertile grains from five panicles taken at random from each yield square were counted to determine the sterility rate. Stem, leaf, and panicle biomass were determined. After 15 days of air-drying the dry mass, 1000 kernel weight, and yield were assessed using an electronic balance.

#### 2.3.2 Soil chemical properties

Soil samples were collected at harvesting stage of rice. Samples were collected from the 0–20, 20–40, 40–60, 60–80, and 80–100 cm soil layers using an auger at each treatment. A composite of five samples was used following a cross-sampling design to determine the soil pH, and EC. Soil samples were taken before and after the application of the treatments. The samples were dried and stored at room temperature for 20 days in the ISRA laboratory and then analyzed. Soil pH_H2O_/_KCL_ was determined using a HANNA pH meter every 15 days until harvest. The EC of the filtrate was measured using a conductivity meter. Soil pH and EC were measured with a 1:2.5 and 1:5 soil-to-water ratio respectively. The pH_KCL_ was determined by adding 3.75 g of KCL to the pH_H2O_ suspension.

### 2.4 Data analyses

Two-way analyses of variances (ANOVAs) for growth and yield parameters and soil chemical properties were performed with R 4.1.3 [17]. When the effects were significant, Tukey's test was used for multiple mean comparisons to detect the significant differences between the factors and treatments. Statistical significance was fixed at 0.05. Considering the cultural mode, fertilizers, and soil depth, all data are hence expressed as overall means ± SE. Clustering based on bray ecological distance and principal components analyses were done to study the relationships between agro-morphological parameters, cultural mode, fertilizers, and treatments. Clustering analyses were done using BioversityR package [18].

## 3 Results

### 3.1 Growth parameters

**Fig. 3:**
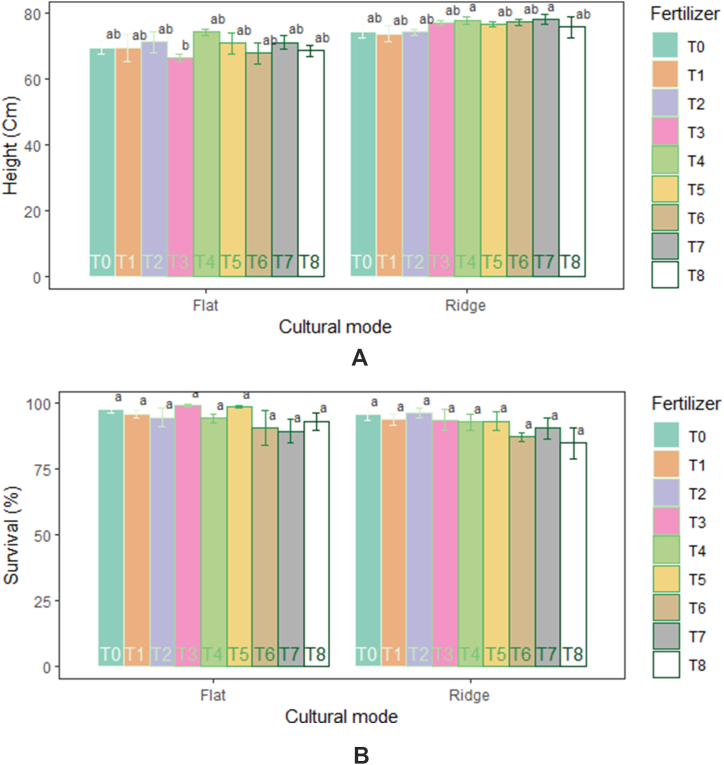
Changes in height (A) and survival (B) of rice in response to cultural mode and fertilizers. Values are means ± SE; significant differences are indicated with different letters.

Cultural mode influenced significantly the height (p = 2.56e-07) of rice plants. Higher plant height (76.26 cm) was recorded in the ridge. Furthermore, no significant impact of fertilizer on growth parameters was recorded (Table 2). There was no combined effect of cultural mode and fertilizer on growth parameters, except for the height of plants resulted in significantly higher values in ridge mode associated with 7.5 t ha^−1^ compost +100 kg ha^−1^ NPK +75 kg ha^−1^ N (78.31 cm) and 5 t ha^−1^ compost + 100 kg ha^−1^ NPK + 75 kg ha^−1^ N (8.06 cm). In absolute value, the higher survival rates were recorded in amendment application of control (97.57%), 5 t ha^−1^ compost (98.96%), and 5 t ha^−1^ compost + 200 kg ha^−1^ NPK +150 kg ha^−1^ N (98.61%) under flat mode (Fig. 3 (A, B)).

**Table 2:** Variation of growth and yield parameters and yield according to fertilizers.

Fertilizers	Growth parameters		Yield parameters				Yield		
	Survival (%)	Height (cm)	Tillers (m^−2^)	Panicules (m^−2^)	Sterility (%)	Infertility (%)	1000 kernel weight (g)	Yield (kg ha^−1^)	Biomass (g)
T0	96.53 ± 1.93^a^	72.19 ± 2.41^a^	66.21 ± 12.15^a^	45.70 ± 13.55^a^	48.84 ± 8.10^a^	34.08 ± 11.68^a^	13.50 ± 0.55^a^	797.00 ± 280.20^a^	2195.75 ± 382.62^b^
T1	94.79 ± 1.81^a^	71.69 ± 3.44^a^	98.63 ± 25.53^a^	55.47 ± 13.70^a^	44.98 ± 9.54^a^	35.37 ± 16.41^a^	13.87 ± 0.92^a^	816.75 ± 201.04^a^	2897.28 ± 263.25^ab^
T2	95.31 ± 2.68^a^	72.86 ± 2.38^a^	86.13 ± 9.88^a^	60.74 ± 11.00^a^	53.47 ± 10.36^a^	30.96 ± 6.47^a^	13.49 ± 0.58^a^	731.25 ± 177.35^a^	2988.25 ± 352.71^ab^
T3	96.18 ± 3.01^a^	71.73 ± 2.99^a^	73.83 ± 10.97^a^	58.79 ± 12.41^a^	44.73 ± 6.96^a^	21.77 ± 7.79^a^	13.08 ± 0.72^a^	764.50 ± 272.83^a^	2842.88 ± 216.01^ab^
T4	93.40 ± 2.37^a^	76.23 ± 1.36^a^	87.70 ± 14.71^a^	68.55 ± 14.28^a^	53.80 ± 11.30^a^	22.83 ± 5.64^a^	12.14 ± 1.11^a^	834.75 ± 268.83^a^	3188.00 ± 208.94^ab^
T5	95.83 ± 2.83^a^	73.91 ± 2.70^a^	86.13 ± 4.19^a^	51.56 ± 12.93^a^	59.65 ± 11.40^a^	41.30 ± 13.81^a^	12.15 ± 1.21^a^	835.50 ± 302.37^a^	3640.25 ± 330.77^a^
T6	88.89 ± 4.56^a^	72.67 ± 3.31^a^	68.75 ± 9.70^a^	45.31 ± 12.75^a^	52.06 ± 9.80^a^	36.49 ± 12.61^a^	11.29 ± 2.08^a^	755.88 ± 334.07^a^	2582.38 ± 552.21^ab^
T7	89.76 ± 3.87^a^	74.78 ± 2.48^a^	75.59 ± 9.87^a^	54.49 ± 14.74^a^	45.77 ± 6.79^a^	31.24 ± 13.93^a^	11.67 ± 2.01^a^	715.00 ± 257.43^a^	3339.25 ± 432.23^a^
T8	88.89 ± 4.97^a^	72.27 ± 3.00^a^	88.28 ± 7.38^a^	54.88 ± 11.82^a^	64.63 ± 10.62^a^	38.21 ± 13.05^a^	11.17 ± 2.08^a^	799.13 ± 281.03^a^	3483.13 ± 424.95^a^
p-value	0.0861	0.491	0.17736	0.382	0.35573	0.55	0.375	0.999	0.0037

T0: control, T1: 100 kg ha^−1^ NPK + 75 kg ha^−1^ N, T2: 200 kg ha^−1^ NPK + 150 kg ha^−1^ N, T3: 5 t ha^−1^ compost, T4: 5 t ha^−1^ compost + 100 kg ha^−1^ NPK + 75 kg ha^−1^ N, T5: 5 t ha^−1^ compost + 200 kg ha^−1^ NPK+150 kg ha^−1^ N, T6: 7.5 t ha^−1^ compost, T7: 7.5 t ha^−1^ compost + 100 kg ha^−1^ NPK + 75 kg ha^−1^ N, T8: 7.5 t ha^−1^ compost + 200 kg ha^−1^ NPK+ 150 kg ha^−1^N. Results are expressed as mean ± SE, letters a and b are groups (groups with different letters are significantly different).

### 3.2 Yield parameters

**Fig. 4:**
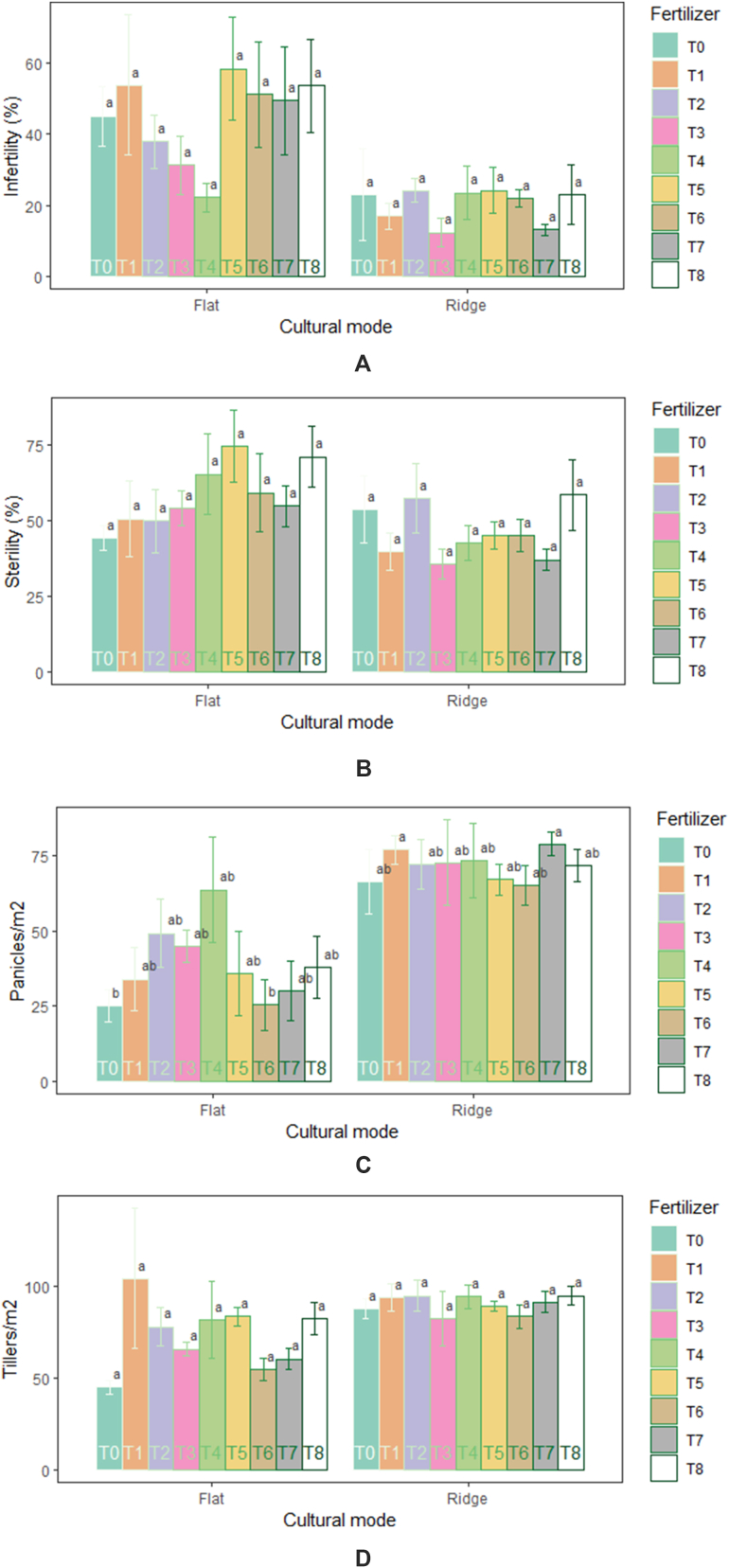
Performance in infertility (A), sterility (B), panicles (C), and tillers (D) of rice in response to cultural mode and fertilizers.

Yield parameters varied significantly (p˂0.05) between cultural modes. Number of Tillers (89.93 m^-2^) and panicles (71.65 m^-2^) were higher in ridge than flat. There were significantly more infertility (44.74%) and sterility (58.04%) at flat than ridge mode. No significant effect of fertilizers on yield parameters was noticed (Table 2). However, the amendment application of 100 kg ha^−1^NPK +75 kg ha^−1^ N, 200 kg ha^−1^NPK +150 kg ha^−1^ N, 5 t ha^−1^ compost +100 kg ha^−1^ NPK +75 kg ha^−1^ N and 7.5 t ha^−1^ compost +200 kg ha^−1^ NPK +150 kg ha^−1^N produced in absolute value more tillers and panicles. The results showed no significant interaction (cultural mode and fertilizer) effect (p˃0.05) on infertility, sterility, and tillers (Fig. 4 (A-D)), except for the number of panicles which was significantly (p = 0.016) influenced. The number of panicles increased significantly with the amendment application of 100 kg ha^−1^NPK +75 kg ha^−1^N (76.95 m^-2^) and 7.5 t ha^−1^ compost + 100 kg ha^−1^NPK + 75 kg ha^−1^ N (78.91 m^-2^) under ridge mode.

### 3.3 Yield

**Fig. 5:**
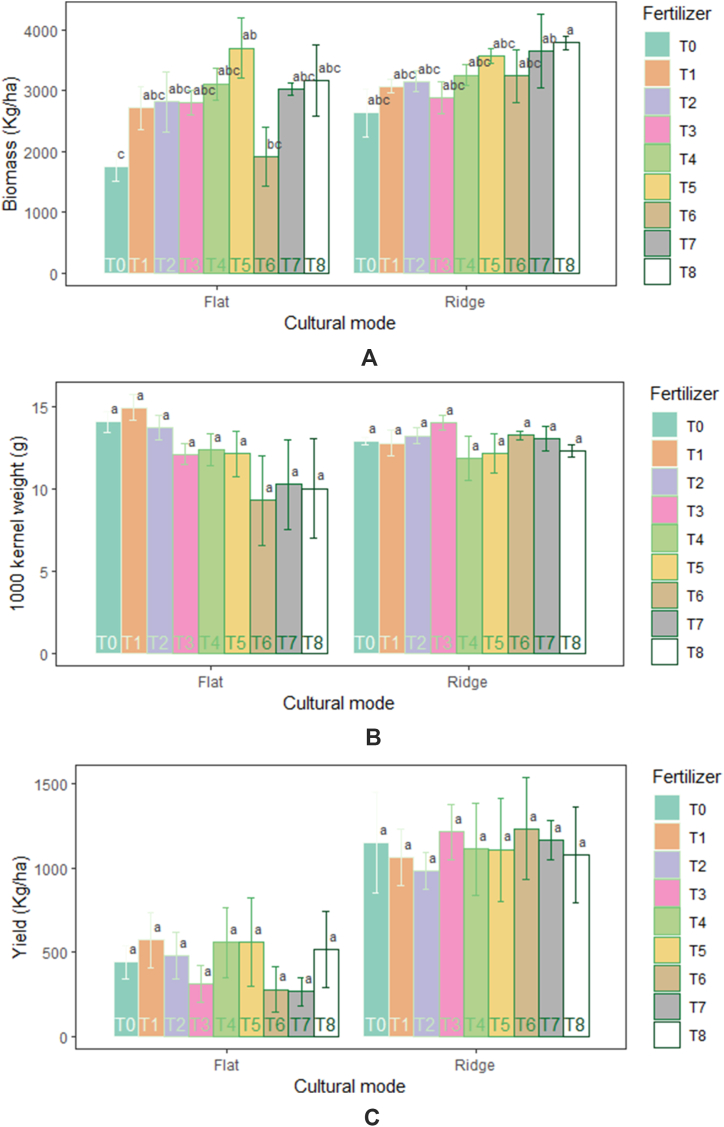
Changes in Biomass (A), 1000 kernel weight (B), and yield (C) in response to cultural mode and fertilizers.

Significant variation (p = 4.24e-09) in the yield of rice was observed under different cultural modes. Higher biomass (3252.25 g), 1000 kernel weight (12.85 g), and yield (1123.14 kg ha^−1^) of rice was recorded in ridge compared to flat mode. Yield and 1000 kernel weight were not significantly (p˃0.05) affected by fertilizer. But biomass varied significantly (p = 0.0037) between fertilizer types (Table 2). Biomass was greater in amendment application of 5 t ha^−1^ compost +200 kg ha^−1^ NPK +150 kg ha^−1^ N (3640.25 g), 7.5 t ha^−1^compost + 100 kg ha^−1^NPK + 75 kg ha^−1^ N (3339.25 g), and 7.5 t ha^−1^ compost + 200 kg ha^−1^NPK + 150 kg ha^−1^ N (3483.12 g) than in control (2195.75 g). The interactions (cultural mode and fertilizers) had no significant effect (p˃0.05) on yield, except for only the biomass (Fig. 5 (A-C)). Biomass recorded in amendment application of 7.5 t ha^−1^ compost +200 kg ha^−1^NPK +150 kg ha^−1^ N (3790.75 g) under ridge mode was significantly higher than those in control (1754 g) and 7.5 t ha^−1^ compost (1922.25 g) under flat mode.

### 3.4 Soil chemical properties

**Fig. 6:**
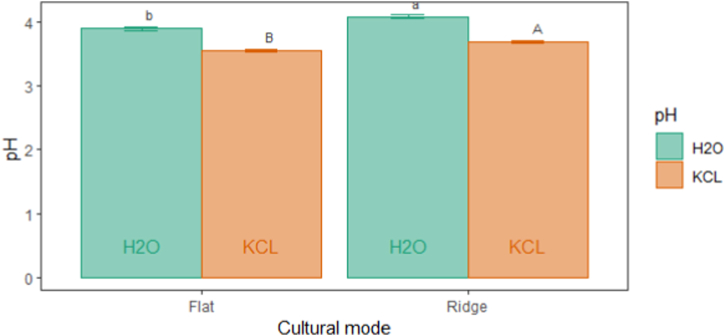
Changes in pH_H2O_ and pH_KCl_ in response to cultural mode.

**Fig. 7:**
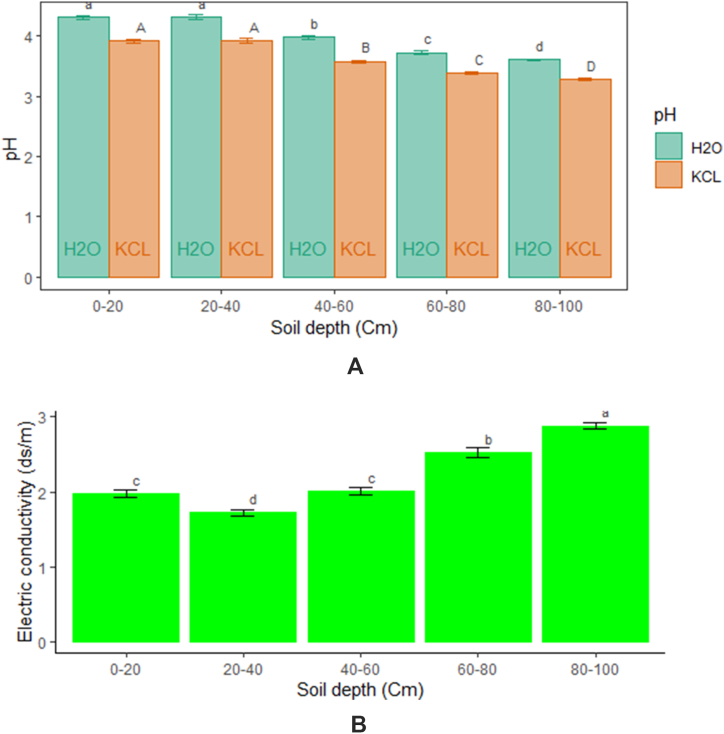
Changes in Soil pH (A) and EC (B) according to soil depth.

Significance differences (p˂0.05) were observed in soil pH and salinity for all two cultural modes (Fig. 6). Both cultural modes presented low soil pH (3.53–4.08) and high electrical conductivity (2.13–2.31 ds m^−1^). A strong variation of pH and EC between cultural modes was characterized by higher pH_H2O_ (4.08) and pH_KCL_ (3.68) in ridge mode than for flat. The soil EC (2.13 ds m^−1^) was significatively lower in ridge mode than for flat (2.31 ds m^−1^). Soil depth level influenced significantly (p˂0.05) soil chemical properties. A decrease in pH and an increase in EC from topsoil to depth were observed (Fig. 7 (A, B)). The pH_H2O_ (4.31) and pH_KCL_ (3.92) were significantly higher in topsoil than in depth. EC increased significantly from topsoil (1.73 ds m^−1^) to depth (2.88 ds m^−1^). The fertilizers influenced also significantly (p˂0.05) soil pH and EC (Table 3). The soil pH_H2O_ (4.08) and pH_KCL_ (3.72) were significantly higher in 7.5 t ha^−1^ compost + 200 kg ha^−1^ NPK+ 150 kg ha^−1^N than in 5 t ha^−1^ compost + 200 kg ha^−1^ NPK+150 kg ha^−1^ N (3.91 and 3.53) and 200 kg ha^−1^ NPK + 150 kg ha^−1^ N (3.94 and 3.57). Fertilizer “100 kg ha^−1^ NPK +75 kg ha^−1^ N “decreased significantly EC (2.02 ds m^−1^) than for 7.5 t ha^−1^ compost (2.36 ds m^−1^) and 7.5 t ha^−1^ compost + 100 kg ha^−1^ NPK + 75 kg ha^−1^ N (2.34 ds m^−1^). Soil pH and salinity varied significantly between cultural mode and fertilizers (Appendix A). The organo-mineral and organic fertilizers under ridge mode increased more soil pH. However, mineral fertilizers reduced significantly salinity.

**Table 3:** Variation of soil pH and salinity according to fertilizers.

Fertilizers	Soil chemical proprieties		
	pH_H2O_	pH_KCl_	EC (ds m^−1^)
T0	3.99 ± 0.06^ab^	3.61 ± 0.05^abc^	2.27 ± 0.10^ab^
T1	4.01 ± 0.06^ab^	3.61 ± 0.05^abc^	2.02 ± 0.08^b^
T2	3.94 ± 0.05^b^	3.57 ± 0.05^bc^	2.13 ± 0.10^ab^
T3	3.97 ± 0.05^ab^	3.58 ± 0.05^bc^	2.16 ± 0.10^ab^
T4	4.02 ± 0.07^ab^	3.65 ± 0.06^abc^	2.24 ± 0.09^ab^
T5	3.91 ± 0.05^b^	3.53 ± 0.04^c^	2.16 ± 0.10^ab^
T6	4.02 ± 0.07^ab^	3.67 ± 0.07^ab^	2.37 ± 0.11^a^
T7	3.96 ± 0.06^ab^	3.60 ± 0.05^abc^	2.34 ± 0.09^a^
T8	4.08 ± 0.07^a^	3.72 ± 0.07^a^	2.32 ± 0.09^ab^
p-value	0.00629	0.000229	0.00823

### 3.5 Relationship between growth and yield parameters, yield, fertilizer, and cultural mode

**Fig. 8:**
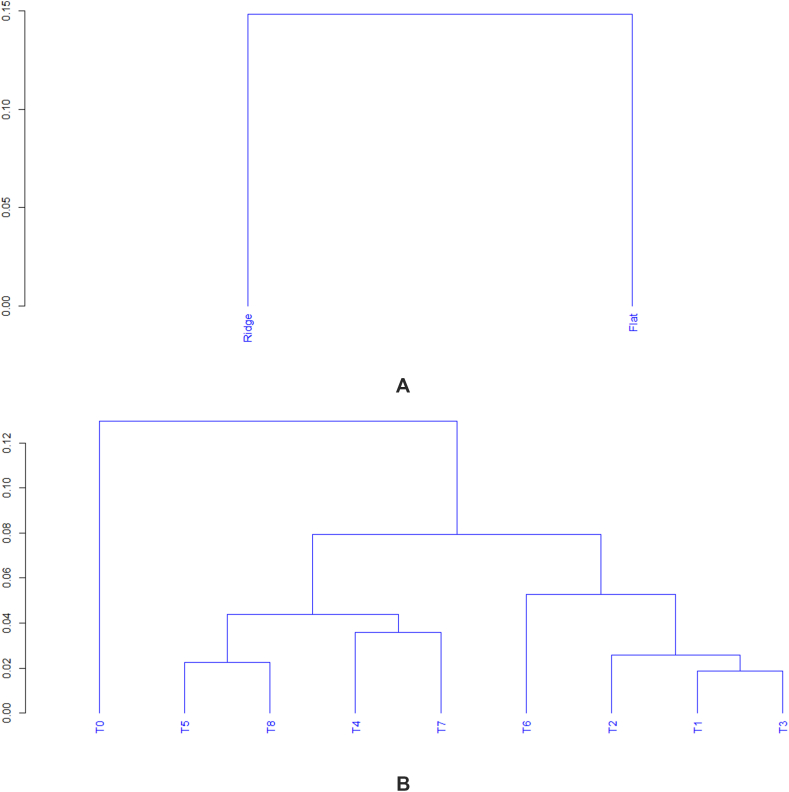
Cluster dendrogram of cultural mode (A) and fertilizers (B).

**Fig. 9:**
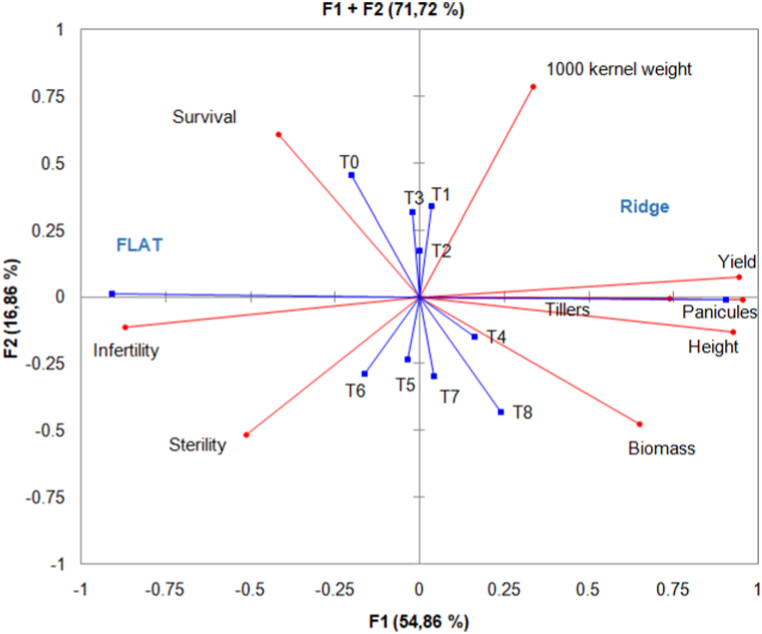
Relationship between growth and yield parameters, yield, fertilizers, and cultural mode.

Clustering combined with Principal component Analysis (PCA) on qualitative and quantitative parameters showed groups according to cultural mode and fertilizers (Fig. 8 (A, B)). For the cultural mode, the analysis separated two distinct groups (ridge and flat). The first group was flat mode characterized by higher survival, infertility, and sterility values. The second group (ridge mode) was characterized by improved growth and yield parameters increasing the yield of rice. Analysis-based fertilizers discriminated four groups according to their influence on growth and yield parameters and yield of rice. The control was a separate group with high survival. The fertilizer with 7.5 kg ha^−1^ compost constituted a group associated with high biomass production, infertility, and sterility. The group of organo-mineral fertilizers was characterized by higher performance in tillers, panicles, height, biomass, and yield of rice. The last group was mineral and organic fertilizers with relatively good tillers and panicles production, 1000 kernel weight, and yield. PCA showed a correlation between yield and growth parameters (tillers, panicles, and height) and yield (100 kernel weight, biomass, and yield). Improved yield and parameters increased the yield of rice. A strong correlation between infertility and sterility was noticed (Fig. 9).

## 4 Discussion

### 4.1 Influence of cultural modes and fertilizers on growth and yield parameters and yield of rice

The results showed that cultural mode influenced the growth and yield parameters. Compared to flat, ridge mode induced an increase in height, tillers, panicles, 1000 kernel weight, yield, and biomass of rice. However, flat mode increased significantly survival, infertility, and sterility of the rice compared to ridge mode. The use of ridges resulted in the highest number of tillers per plant and a lower percentage of unfilled grains than flat cultivation [19]. Maximum plant height, grain weight, and 1000 kernel weight of rice were measured in bed planting compared to flat sowing [20]. Ridge mode changed soil microtopography and was considered a version of ‘semi-upland’ tillage regimes [21]. The ridges formed by the ridge tillage were completely above the water surface, which reduced the contact between water and fertilizers, thereby reducing potential for runoff and leaching loss of nutrients [21].

Beneficial effects of the application of fertilizers on rice crop growth (growth and yield parameters and yield) were observed in the treatments over the control. Fertilizer applications showed no significantly difference on growth and yield parameters. Nutrient management practices showed no significantly difference on number of panicles per unit area during the rice cultivation [22]. However, Organic and organo-mineral fertilizers induced a relative increase in growth and yield parameters compared to other fertilizers (control and mineral). The values obtained for biomass production differed significantly between fertilizers. Organo-mineral fertilizers increased significantly the biomass. The benefit of using organic fertilizers was due to release of aliphatic and aromatic hydroxyl acids and humates leaded to higher availability of nutrients [22]. NPK and organo-mineral combined effects significantly influenced the plant height and number of leaves compared to no fertilizer (control) [23]. The greatest increase in growth parameters including plant height (97.5 cm), number of tillers (325.5 m^-2^), number of panicles (308.5 m^-2^), and 1000-grain weight (24.0 g) of rice occurred with the application of organic and organo-mineral fertilizers over the control and other fertilizers [24]. Under the application of different fertilizer types, inorganic fertilizers produced the highest number of tillers per plant and grain yield of rice followed by organic fertilizers and control [19]. Fairhurst et al. [25] found that the application of N and P nutrients resulted in an increase in the numbers of tillers and panicles, panicle length (cm), and the number of spikelets and consequently an increase in grain yield. According to Inckel et al. [26], organic matter contains important nutrients such as nitrogen (N), phosphorus (P), and potassium (K) which will be available to the plants after decomposition.

Good performance of crops in rice cultural modes may be associated with soil fertility. The combination of cultural mode and fertilizers affected the height, number of panicles, and biomass but the effects on survival rate, infertility, sterility, tillers, 1000 kernel weight, and yield of rice were not significant. Treatments based on the combination of ridge mode and organic and organo-mineral fertilizers performed better than other treatments. Grain weight and yield of rice were higher in organo-mineral than mineral and control [27]. The grain yield of rice and its components were significantly increased in all the treatments over control and the higher grain yield was noted in plants treated with organo-mineral fertilizer [28].

The study showed a correlation between growth and yield parameters (tillers, panicles, and height) and yield (100 kernel weight, biomass, and yield). Panicle number, grain number per panicle, seed setting rate, and grain weight were the main factors of production [29]. It was currently believed that increasing the tiller rate would easily increase crop yield [30]. The cultivation method could impact panicle number [31].

### 4.2 Influence of cultural modes and fertilizers on soil chemical properties

Initially, the experience was carried in acid sulphate soils. These soils were characterized by low pH varying between 4.08 and 3.53 and high salinity. To ameliorate and regulate soil acidity, nutrients and salinity, cultural mode and fertilizer application were important practices adopted in many parts of the world. There was a significant improvement of soil pH and EC due to cultural modes and the application of fertilizers. Compared with soil chemical properties under cultural modes, ridge mode increased soil pH and reduced salinity significantly. This might be due to the fact that ridge mode, created better physico-chemical conditions, which enhance the soil microbial activities and fertility. Rice ridge tillage was designed to improve the soil condition, mitigate drought stress, decrease water consumption, and increase yield by constructing a mixed water and dry binary environment using agricultural machinery. Ridge tillage only differed from others by operationally introducing mechanized dry farming ridging techniques. Moreover, ridge tillage could enhance compaction of rice soil, provide structure to dry farming soils, and improve soil respiration [32]. Soil pH and EC were also influenced by fertilizers. Among fertilizers, the organo-mineral fertilizers increased soil pH and ensured sufficient availability of nutrients in the soil while mineral fertilizers decreased the salinity. The soil pH values obtained in organic and organo-mineral were significantly higher than in the control and mineral. In acid sulphate soils, organic matter has a greater alkalizing impact than inorganic salts. The application of organic compounds would have increased the soil pH in acid sulphate soils [33]. Liming enhances the physical, chemical and biological properties of acid soils [34]. The low pH value obtained with the inorganic fertilizer application might be due to the acid-yielding property of urea fertilizer served as the source of starter N [35]. Long-term application of organic compost in low-pH soil increased pH compared to treatments receiving chemical fertilizer [36,37]. Butler and Muir [38] reported that the soil pH increased with organic compost application. In the long-term organic compost-added experiment, Walker et al. [39] also recorded the higher pH in acidic soils with repeated application of organic compost. The decrease in EC for the organic compost-amended soil may be due to an increase in the leaching of water-soluble salts into the subsoil [38], because of improvement in soil physical properties [40]. The application of gypsum also had a significant effect on soil salinity. However, there were non-significant differences among different forms of gypsum applied to the soil [41]. Soil amendments (chemical) typically reduce salinity but have great variability in yield increase [42].

Soil chemical properties varied according to depth. The soil pH decreased from topsoil to depth while EC increased. A decrease in soil pH and an increase in EC with increasing soil depth were observed [43]. However, higher soil salinity (11.2 dS m^−1^) in the surface soil layer of acid sulphate soils of Ganges Delta was also reported [4]. Soil salinization in Basse Casamance occurs through the capillary rise of the saline water table in the profiles [44]. But authors found that the salinity gradient was vertically downwards and Subsoil salinity was usually much lower than topsoil salinity [45,46]. Saltwater intrusion from shrimp ponds and brackish water aquaculture into agricultural land has widely been recognized [47].

## 5 Conclusion

Rice cultivation requires good practices and adequate soil conditions for better yields under acid sulphate soils. This study on the effects of cultural mode and fertilizers on rice growth and yield, and soil pH and salinity showed that cultural mode influenced rice performance. Ridge cultivation favored rice performance while improving soil chemical properties (increasing soil pH and decreasing soil salinity). The various mineral, organic and organo-mineral fertilizers substantially improved rice yield and soil chemistry. Both organo-mineral and organic fertilizers significantly increased rice yield. Ridge mode combined with organo-mineral amendment improved rice performance and soil chemical properties. Fertilizer mode and type are critical elements to improve soil pH, salinity, and yield.

## Funding

This research was carried out in KOPIA Project funded by Korean Program on International Agriculture (KOPIA).

## Author contribution statement

All authors listed have significantly contributed to the conception, design and performance of the experiments, the analysis and interpretation of data and the writing of this article.

## Data availability statement

Data will be made available on request.

## Declaration of competing interest

All of the authors declare that they have all participated in the design, execution, and analysis of the paper, and that they have approved the final version. Additionally, there are no conflicts of interest in connection with this paper, and the material described is not under publication or consideration for publication elsewhere.

## Figures and Tables

**Table:** 

Mode	Soil depth (Cm)	pH_H2o_								
		T0	T1	T2	T3	T4	T5	T6	T7	T8
Ridge	0-20	4.22±0.16^ab^	4.30±0.16^ab^	4.15±0.08^bc^	4.09±0.08^cd^	4.20±0.16^ab^	4.14±0.09^bc^	4.48±0.18^ab^	4.17±0.08^ab^	4.48±0.19^ab^
	20-40	4.46±0.08^ab^	4.43±0.12^ab^	4.44±0.16^ab^	4.36±0.07^ab^	4.74±0.15^a^	4.33±0.08^ab^	4.58±0.16^ab^	4.46±0.08^ab^	4.69±0.22^ab^
	40-60	4.21±0.11^ab^	4.14±0.13^bc^	4.09±0.10^cd^	4.11±0.10^bc^	4.21±0.04^ab^	4.02±0.10^cd^	4.23±0.14^ab^	4.11±0.07^bc^	4.31±0.13^ab^
	60-80	3.85±0.14^ij^	3.86±0.16^gh^	3.85±0.10^ij^	3.81±0.09^jk^	3.84±0.03^jk^	3.79±0.10^jk^	3.87±0.07^fg^	3.81±0.04^jk^	3.97±0.07^de^
	80-100	3.71±0.08^op^	3.69±0.11^op^	3.65±0.05^pq^	3.64±0.07^pq^	3.65±0.02^pq^	3.64±0.05^pq^	3.62±0.06^rs^	3.64±0.04^pq^	3.72±0.04^mn^
Flat	0-20	4.34±0.15^ab^	4.31±0.05^ab^	4.12±0.05^bc^	4.47±0.11^ab^	4.53±0.17^ab^	4.17±0.07^ab^	4.46±0.13^ab^	4.44±0.06^ab^	4.58±0.06^ab^
	20-40	4.16±0.05^ab^	4.27±0.15^ab^	4.07±0.05^cd^	4.10±0.07^cd^	4.22±0.07^ab^	4.05±0.03^cd^	4.14±0.05^bc^	4.13±0.09^bc^	4.07±0.14^cd^
	40-60	3.78±0.03j^k^	3.91±0.05^ef^	3.85±0.06^hi^	3.83±0.10^jk^	3.72±0.11^no^	3.85±0.04^ij^	3.77±0.03^kl^	3.73±0.07^lm^	3.83±0.11^jk^
	60-80	3.60±0.5^st^	3.62±0.04^qr^	3.65±0.07^pq^	3.70±0.09^op^	3.57±0.05^uv^	3.57±0.04^tu^	3.59±0.03^st^	3.56±0.03^vw^	3.58±0.04^tu^
	80-100	3.53±0.05^yz^	3.53±0.07^yz^	3.56±0.09^uv^	3.60±0.05^st^	3.55±0.03^wx^	3.54±0.03^wx^	3.52±0.02^z^	3.54±0.02^xy^	3.56±0.03^vw^
		pH_KCL_								
Ridge	0-20	3.87±0.14^ab^	3.90±0.13^ab^	3.56±0.14^ef^	3.74±0.07^bc^	3.85±0.13^ab^	3.77±0.11^bc^	4.13±0.14^ab^	3.83±0.08^ab^	4.15±0.20^ab^
	20-40	3.98±0.09^ab^	3.96±0.12^ab^	4.02±0.13^ab^	3.93±0.05^ab^	4.23±0.13^ab^	3.90±0.09^ab^	4.19±0.16^ab^	4.11±0.02^ab^	4.34±0.23^a^
	40-60	3.72±0.07^bc^	3.71±0.12^bc^	3.73±0.08^bc^	3.65±0.09^cd^	3.74±0.04^bc^	3.61±0.08^de^	3.79±0.13^ab^	3.65±0.07^cd^	3.86±0.11^ab^
	60-80	3.49±0.10^fg^	3.50±0.11^fg^	3.45±0.06^gh^	3.40±0.04^ij^	3.42±0.02^ij^	3.41±0.08^ij^	3.40±0.07^jk^	3.35±0.04^kl^	3.65±0.12^cd^
	80-100	3.34±0.08^lm^	3.32±0.08^mn^	3.27±0.04^op^	3.25±0.07^qr^	3.28±0.04^op^	3.26±0.08^pq^	3.30±0.04^no^	3.34±0.07^lm^	3.44±0.09^hi^
Flat	0-20	3.96±0.13^ab^	3.85±0.04^ab^	3.82±0.10^ab^	4.01±0.14^ab^	4.11±0.17^ab^	3.83±0.08^ab^	4.03±0.11^ab^	4.05±0.06^ab^	4.11±0.03^ab^
	20-40	3.75±0.03^bc^	3.82±0.11^ab^	3.76±0.11^bc^	3.74±0.06^bc^	3.78±0.08^bc^	3.65±0.02^cd^	3.99±0.24^ab^	3.77±0.06^bc^	3.67±0.10^cd^
	40-60	3.42±0.02^ij^	3.53±0.03^fg^	3.49±0.06^fg^	3.41±0.04^ij^	3.44±0.06^gh^	3.45±0.03^gh^	3.42±0.04^ij^	3.36±0.07^kl^	3.44±0.08^gh^
	60-80	3.28±0.05^op^	3.33±0.03^mn^	3.35±0.09^kl^	3.34±0.11^kl^	3.33±0.03^mn^	3.25±0.04^qr^	3.24±0.02^qr^	3.31±0.05^mn^	3.30±0.05^no^
	80-100	3.25±0.06^qr^	3.23±0.07^rs^	3.26±0.09^pq^	3.30±0.06^no^	3.28±0.02^op^	3.22±0.02^st^	3.20±0.02^t^	3.24±0.01^qr^	3.28±0.04^op^
		EC (ds/m)								
Ridge	0-20	1.79±0.28^cd^	1.99±0.11^bc^	1.95±0.24^bc^	1.84±0.16^bc^	1.95±0.21^bc^	1.86±0.14^bc^	2.11±0.17^ab^	2.14±0.17^ab^	2.13±0.17^ab^
	20-40	1.49±0.07^ij^	1.72±0.14^ef^	1.58±0.37^hi^	1.64±0.14^fg^	1.76±0.08^de^	1.65±0.12^fg^	1.85±0.25^bc^	1.68±0.08^ef^	1.92±0.15^bc^
	40-60	1.77±0.20^cd^	1.70±0.27^ef^	1.65±0.36^fg^	1.83±0.26^bc^	1.82±0.20^cd^	1.72±0.19^ef^	1.98±0.29^bc^	1.76±0.16^cd^	1.97±0.28^bc^
	60-80	2.42±0.26^ab^	2.10±0.29^ab^	2.22±0.26^ab^	2.41±0.31^ab^	2.45±0.11^ab^	2.42±0.32^ab^	2.58±0.29^ab^	2.50±0.20^ab^	2.22±0.23^ab^
	80-100	2.71±0.19^ab^	2.62±0.17^ab^	2.62±0.13^ab^	2.73±0.22^ab^	2.79±0.17^ab^	2.60±0.08^ab^	3.39±0.34^a^	2.90±0.14^ab^	2.92±0.15^ab^
Flat	0-20	2.21±0.38^ab^	1.71±0.05^ef^	2.19±0.38^ab^	1.72±0.13^ef^	2.15±0.51^ab^	1.89±0.17^bc^	2.02±0.14^bc^	2.06±0.19^ab^	1.80±0.16^cd^
	20-40	1.84±0.09^bc^	1.41±0.06^j^	1.73±0.24^ef^	1.68±0.07^ef^	1.66±0.11^fg^	1.61±0.19^gh^	1.81±0.10^cd^	1.98±0.12^bc^	2.11±0.33^ab^
	40-60	2.48±0.17^ab^	1.80±0.09^cd^	1.97±0.20^bc^	2.32±0.25^ab^	2.19±0.12^ab^	2.08±0.23^ab^	2.37±0.08^ab^	2.56±0.08^ab^	2.27±0.16^ab^
	60-80	2.91±0.09^ab^	2.40±0.11^ab^	2.69±0.28^ab^	2.32±0.45^ab^	2.79±0.04^ab^	2.83±0.29^ab^	2.43±0.43^ab^	3.00±0.18^ab^	2.73±0.17^ab^
	80-100	3.08±0.07^ab^	2.80±0.26^ab^	2.73±0.16^ab^	3.10±0.24^ab^	2.90±0.13^ab^	2.96±0.24^ab^	3.09±0.10^ab^	2.79±0.36^ab^	3.16±0.09^ab^

## Appendix A

Appendix A: Comparison of soil pH and EC among different treatments and soil layers.
